# Repetitive Grasping After Stroke Assisted by Functional Electrical Stimulation

**DOI:** 10.1111/aor.15033

**Published:** 2025-06-27

**Authors:** Chiara Höhler, Satoshi Endo, Joachim Hermsdörfer, Lucille Cazenave, Hossein Kavianirad, Klaus Jahn, Carmen Krewer

**Affiliations:** ^1^ Department of Neurology—Research Group Schoen Clinic Bad Aibling Bad Aibling Germany; ^2^ Department Health and Sport Sciences, TUM School of Medicine and Health Technical University of Munich Munich Germany; ^3^ Chair of Information‐oriented Control, TUM School of Computation, Information and Technology Technical University of Munich Munich Germany; ^4^ UKRI Centre for Doctoral Training in AI for Healthcare Imperial College London London UK; ^5^ Bioengineering Department Imperial College London London UK; ^6^ German Center for Vertigo and Balance Disorders (DSGZ) Ludwig‐Maximilians‐Universität in Munich Munich Germany

**Keywords:** activities of daily living, assistive devices, Functional Electrical Stimulation, grip force, stroke

## Abstract

**Background:**

Grip force requirements for grasping and releasing objects when hand opening and closing is assisted by Functional Electrical Stimulation (FES) were investigated. To understand whether FES can be useful in assisting these motion primitives, the following requirements were investigated: (i) producing sufficient force, (ii) sustaining this force for an adequate duration, (iii) successfully releasing the object, and (iv) monitoring the onset of muscle fatigue during repetitive grasping.

**Methods:**

In an observational cross‐sectional design, hemiparetic patients after stroke were instructed to repetitively grasp and release a sensorized object at maximum voluntary contraction while receiving FES assistance (3 s each of finger flexor and extensor stimulation). Outcome variables included clinical grip force data of the paretic and non‐paretic hand. Grip force metrics, corresponding to each investigated requirement, such as the maximum applied grip force (GFmax), the amount of time of grip force maintained in the “GFtarget ± 10% window,” and the force level during the assisted release (release ratio), were extracted during the FES‐assisted grasping protocol. Additionally, changes in GFmax across repetitions were analyzed to investigate muscle fatigue.

**Results:**

The median GFmax of 16 analyzed patients (mean 3.4 months post stroke) was 6.9 N (2.2–56.5 N), with severely impaired patients producing significantly lower values than the mildly/moderately impaired. The maximum grip force level was maintained for a median of 1.8 s, and the median object release ratio was significantly higher in patients with spasticity.

**Conclusion:**

All patients were able to produce grip force during FES assistance and maintained the grip force level across repetitions, without physiologically meaningful fatigue. The grip forces produced by the patients were similar to those found in real‐life object handling in healthy subjects and very close to the reference values required by stroke patients for object transportation. In addition, the repetitive grasping did not lead to a significant muscle fatigue. This grip force produced while having no relevant fatigue highlights the assistive potential of FES in activities of daily living. However, these effects need to be verified for the grasping of real‐life objects.

**Trial Registration:**

This trial was registered on September 29, 2021 (registration number: DRKS00025889)

## Graphical abstract

To understand whether FES can be useful in assisting functional grasping after stroke, the following requirements were investigated: (i) producing sufficient force, (ii) sustaining this force for an adequate duration, (iii) successfully releasing the object, and (iv) monitoring the onset of muscle fatigue during repetitive grasping.

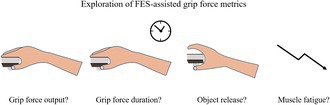

## 1 Background

### 1.1 Rationale

Impairments of the upper limb are the most prevalent symptoms after a stroke, occurring in about 63% of patients (when taking the mean of existing prevalence studies [1]). More than half of these patients are still unable to perform activities of daily living (ADLs) after being discharged from a rehabilitation hospital. Even though one third of patients show improvements in body structures (e.g., strength, range of motion), they do not involve the paretic limb more frequently in ADLs after rehabilitation [2]. Evidence suggests that the longer the time since stroke, the smaller and slower the recovery [3, 4, 5, 6]. This emphasizes the need to explore assistive technologies that can enable and encourage patients with incomplete recovery to make functional use of the paretic limb.

Functional Electrical Stimulation (FES) is a well‐established method that has been used for decades in stroke rehabilitation to regain impaired sensorimotor functions [7]. An electrical current is applied to the peripheral nerve in order to elicit a muscle contraction. This compensates for the impaired top‐down motor control, lost by damage to central motor areas, that would normally transmit the signal to induce movement [8]. The concomitant connection of the electrical stimulation with the goal to accomplish a functional task is referred to as “Functional” Electrical Stimulation [9]. FES treatment can be divided into orthotic or therapeutic applications, depending on the objective to replace or retain function, respectively [10, 11]. A synonym for the orthotic effect is the term *assistive effect* [12], describing the performance gain between unassisted versus assisted task execution. The orthotic use of FES has been implemented for the lower extremities [13], but only rarely for upper extremity functions, most likely due to the greater complexity of hand movements compared to the partially automated movement patterns during walking [14, 15]. Designing a smooth, comfortable and functional upper limb FES system focusing on the orthotic effect remains a challenge and recent work in upper‐limb FES devices primarily consists of feasibility studies or technical reports [16, 17, 18, 19, 20].

Existing assistive devices designed to support paretic hand functions after stroke include robotic gloves [12, 21, 22]. However, these devices are often bulky and can obstruct tactile feedback from the fingertips. Since the development of FES technologies focuses on a therapeutic rather than an orthotic application, the assistive potential of FES is less explored. However, current advancements (e.g., wireless stimulator units or multi‐array electrodes) principally allow an orthotic FES application, and a randomized cross‐over study recently showed that FES has an orthotic effect in ADL‐like object manipulation, particularly in severely impaired patients [23]. In this cross‐over study, the orthotic effect was mostly present for object manipulation which does not require fine motor skills (e.g., 5 cm cube, baseball [24]). FES assistance enabled the patients to transport these objects either fully or partially, with the latter observation meaning that the grip force was not maintained sufficiently long to reach the target place. Therefore, objective measures of grip force and force control for transport are required to characterize FES‐assisted grasping and its potential for use in daily activities. Furthermore, there is no evidence on how suitable FES assistance is in daily life regarding object release and how much change in force output should be expected during repetitive grasping. Thus, there needs to be a better understanding of the extent to which FES meets the requirements for grasping and releasing objects in daily life. Moreover, from a control perspective, these grip force metrics provide a deeper understanding of the dynamics involved in FES‐assisted gripping, which is crucial for making assistance in daily life applications as automatic, accurate, and low‐fatigue as possible.

### 1.2 Objectives

The aim of this clinical trial was to test whether FES‐assisted grasping enables sufficient force production and control to lift, transport, and release the object. Previous work showed that the amount of grip force produced was highly individual, often limited by the patient's tolerance level at higher stimulation intensities [23]. The grip force produced to manipulate objects is a highly informative and sensitive parameter of skilled motor control, as shown in numerous studies of physiological or neurological conditions [25, 26, 27]. On the one hand, the grip force needs to overcome a minimum necessary force that prevents the object from slipping, depending on various characteristics of the object, but also on the actor and the task. On the other hand, unnecessary high grip forces may cause fatigue, damage fragile objects, and impede skilled manipulation. Besides the grasping phase of object manipulation, increased muscle tone could limit the application in terms of failing to open the hand for object release. Therefore, it was investigated whether stimulation of extensor muscles can enable patients with flexor spasticity to release objects. Lastly, many ADLs involve repetitive grasping (e.g., unpacking groceries, buttoning). Due to the non‐physiological recruitment of muscle fibers [28, 29], FES is known to induce muscle fatigue. Few studies have investigated the effect of FES on muscle fatigue, and although some tried to mitigate its effect by changing the stimulation parameters [30, 31], it is still unclear how many FES‐assisted movements can be repeated until signs of muscle fatigue occur.

## 2 Methods

### 2.1 Study Design and Setting

This clinical trial was conducted as an observational cross‐sectional study. Between October 2021 and the end of April 2022, experimental sessions took place at the Schoen Clinic Bad Aibling, Germany. The trial is reported following the STROBE checklist for cross‐sectional studies.

### 2.2 Participants

All patients after stroke who were admitted to the Schoen Clinic Bad Aibling were screened for eligibility. Prior documentation by therapists and physicians was used to screen patients based on the following inclusion criteria and the study‐ and device‐related exclusion criteria. Patients above the age of 18 years with a diagnosis of an ischemic or hemorrhagic stroke had to be cognitively able to follow instructions, to report a low level or no pain in the wrist or fingers of the paretic limb (Numeric Rating Scale [NRS] < 4), to show functional impairments in the wrist or fingers (Medical Research Council [MRC] Scale ≤ 4), to not exceed a defined level of spasticity in the paretic limb (Modified Ashworth Scale [MAS] ≤ 3), and to be able to sit in a chair for the duration of the session (about 1 h). Pregnancy, somnolence, and severe psychiatric disorders were study‐related exclusion criteria, as were the absence of sensitivity in fingers or wrist, and a failure to elicit finger movements with FES in pre‐tests (e.g., due to atrophy or peripheral neuropathy). Patients were not included if one or more of the device‐related exclusion criteria applied, such as active implantable devices (e.g., pacemaker), other metal implants within the stimulated area, epilepsy, cancer, and wounds in the application area of the electrodes or measuring equipment.

### 2.3 Data Sources

Participants attended one experimental session in which grip force data was collected while they performed FES‐assisted grasping movements with their paretic hand. In addition, demographic data and MAS values were taken from the patients' records. Clinical data (i.e., MRC of the wrist extensors and flexors, maximum (unassisted) grip strength of the paretic and non‐paretic hand using the MAP 130K1 hand dynamometer (KERN & SOHN GmbH, Germany), handedness) were assessed once, no more than 1 week prior to the experimental session.

The experimental session included a repetitive grasping protocol, in which participants were requested to grasp a cuboid‐shaped sensorized object with maximum voluntary contraction while FES support was provided. Grasps were performed at maximum voluntary contraction without lifting the object to explore the potential of FES assistance in maximum force production, independent of the proximal arm function and the object's weight and size. Grip force was measured with a sampling rate of 128 Hz and saved for offline analysis. Wrist and finger extension and flexion movements were stimulated using the Fesia Grasp system (Fesia Technology S.L., Spain), a wireless multi‐array electrical stimulator with electrodes covering distal and dorsal aspects of the participant's affected forearm (see [23] for a more detailed description). Fatigue‐resistant stimulation settings were chosen, with a stimulation frequency of 35 Hz and a pulse width of 250 μs [32, 33, 34]. Out of 32 cathodes, the required electrodes were activated and configured for finger flexion and extension using a tablet app in order to stimulate hand closure and opening. Different stimulation amplitudes could be defined for each of the activated electrodes. Before starting the grasping protocol, the stimulation amplitude was individually set based on the intensity and stimulation pattern required to generate the best grasping and extension motion possible (in terms of force production and direction of movement), considering the patient's maximum tolerated stimulation intensity. This intensity was maintained throughout the whole session. The stimulation protocol of 20 repetitive cycles of grasping and opening was manually started by the assessor when the patient was ready to perform the task.

**FIGURE 1:**
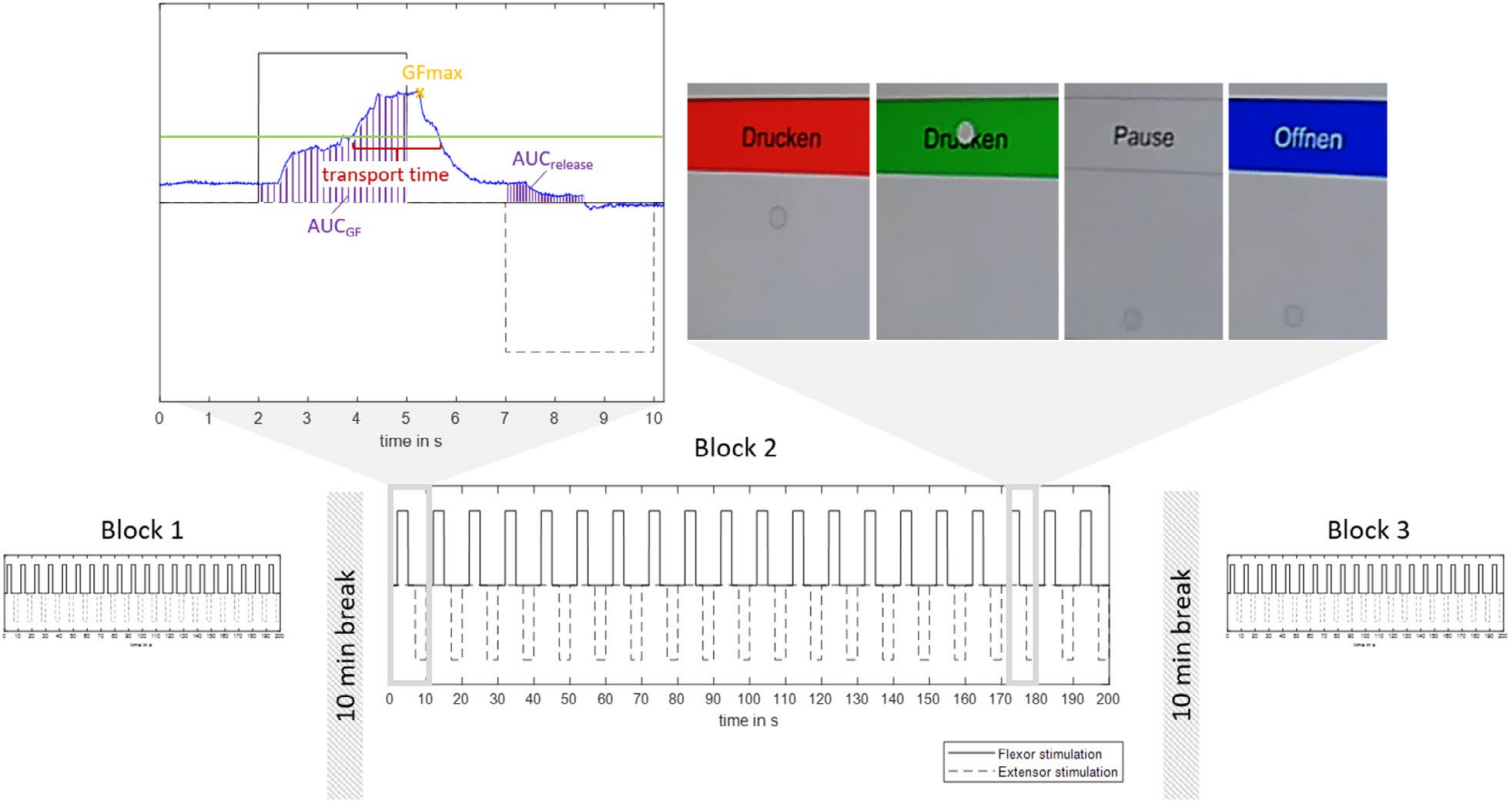
Visualization of the testing protocol, including three identical blocks of 20 repetitions of finger flexion stimulation (solid square waves) followed by finger extension stimulation (dashed square waves). Blocks were separated by a 10‐min break. On the left, the analyzed outcome variables are introduced by an exemplary grip force application in blue and the lower level of GFtarget in green. GFmax: Peak of grip force within one repetition, transport time: Amount of time grip force was above lower level of target window, AUC_GF_: Area under the curve during flexor stimulation, AUC_release_: Area under the curve during extensor stimulation. On the right, the instructions of the graphical user interface, displaying the applied force by means of the gray dot and the target force window in different colors, depending on the current grasping sequence: (A) “Drücken” in red (German for “Squeeze”), (B) “Drücken” in green once the target is reached, (C) “Pause” without stimulation, and (D) “Öffnen” in blue (German for “Open”). Some patients produced substantially higher grip forces during the sequence than during the initial trials, which served to define GFtarget. [Color figure can be viewed at wileyonlinelibrary.com]

While receiving FES assistance, the patients applied grip force to a sensorized object with the thumb in opposition to the other fingers, without lifting the object. The sensorized object was a wireless device (71 × 57 × 22 mm) that records the grip force applied along the depth axis and transmits data via Bluetooth to a PC. The grasping protocol included 3 blocks of 20 repetitions each, with one repetition including 3 s of grasping, 2 s of rest, 3 s of extension, and another 2 s of rest (Figure 1). Breaks of 10 min were scheduled between blocks to provide a sufficient recovery period for the potentially fatigued muscles [35]. Throughout the protocol, the object was taped to a table to prevent it from falling over. The Fesia Grasp stimulator was used with its commercially available software and hardware, and thus with the pre‐set timing of the stimulation (3 s on, 2 s off), kept constant for each participant.

The target force of each repetition was set individually for each patient, based on the FES‐assisted maximum grip force (GFtarget) that was applied to the device. GFtarget was kept constant throughout the whole grasping protocol. To define GFtarget, the patient grasped the object three times as forcefully as possible with concurrent FES stimulation, which was set prior to the start of the experimental protocol. The mean value of this trial was taken as the target value for each of the repetitions, and a target window of ±10% of the average GFtarget was defined. The patient's current grip force, the target window, and the instruction (close—relax—open—relax) were visualized on a monitor, using a Matlab‐programmed Graphical User Interface (GUI, Figure 1).

### 2.4 Bias

Selection bias was reduced by screening all patients who were admitted to the Schoen Clinic Bad Aibling within the recruitment period and by asking all the eligible subjects for their consent to participate. To ensure standardized data collection, the testing procedure was defined a priori (trial registration number: DRKS00025889) and a grasping protocol was followed, guiding all patients through the experiment in the same way by means of the Matlab‐programmed GUI. Within the analysis of muscle fatigue, analyzing changes in grip force within subjects and not between subjects makes individual factors (e.g., attention, daytime, sleep quality) less impactful.

### 2.5 Study Size

A previous sample size calculation estimated 10 participants to be sufficient to detect statistically significant changes in grip force due to neuromuscular fatigue within one testing session [36]. To account for potential dropouts or other factors influencing the availability of patients (spontaneous change in COVID‐19 regulations, no motion resulting from FES) and data (e.g., difficulties in handling data), the aim was to recruit 20 patients.

### 2.6 Quantitative Variables

In order to evaluate the grip force requirements for object manipulation, several grip force parameters were analyzed including (1) the application of sufficient grip force to lift objects, (2) sustaining this force for a time interval sufficient for object transportation, and (3) the ability to release the object; the following grip force parameters were analyzed. Firstly, *GFmax* (Figure 1) of each repetition was inspected and, as a reference, evaluated in relation to the peak grip force exerted by young healthy subjects (lowest value within the group) to lift different ADL objects (as reported in [37]). Secondly, the duration for which a patient can maintain the FES‐assisted force above the lower boundary of the target window was calculated, considering only repetitions where the target window was reached. This time is referred to as *transport time* (Figure 1). As a reference, using the paretic hand, patients after stroke require up to 2 s to transport an object between two targets that are 30 cm apart [25]. Thirdly, the *release ratio* was analyzed to check whether the object was released during stimulation of extensor muscles. For the analysis of the release ratio, the grip force integral during finger extension (AUC_release_; Figure 1) was used, normalized by the grip force applied during finger flexion stimulation (AUC_GF_; Figure 1). In principle, the grip force integral during the time in which finger extensors were stimulated is zero (± noise of the sensorized object).

Another outcome of interest is muscle fatigue. Muscle fatigue is defined as a decline in the produced force by at least 30% [38]. Therefore, the change in GFmax was analyzed. To see if there was muscle fatigue from the beginning to the end of the experiment, the *force drop* was calculated according to the following equation [39, 40]: $\left(1-\frac{\;\mathrm{end}\;\max}{\text{initial}\;\max}\;\right)\times 100$. Hereby, end max was the average GFmax of the last three repetitions while the initial three repetitions are averaged in initial max. Similarly, this metric was calculated for each block of 20 repetitions.

Subgroup analyses were performed for the presence of flexor spasticity and according to the severity of the paresis. The presence of flexor spasticity was defined by a MAS value above zero. The severity of the paresis was categorized into severe and mild/moderate, based on the MRC at wrist (mild/moderate: sum of flexor and extensor score > 5 [1]) and the unsupported grip strength ratio between the paretic and non‐paretic hand (mild/moderate > 0.26 [41]). In case both measures led to an incongruent grouping, the presence or absence of cognitive deficits labeled the patient as severely or mildly/moderately impaired [23], respectively, since motor performance is influenced by cognitive impairments [42].

### 2.7 Statistical Methods

All outcomes of interest were descriptively analyzed and compared to reference values from the literature. In order to see the effect of stimulation on flexor spasticity, the release ratio was correlated with the repetitions performed.

In contrast to patients with a severe paresis, there is residual motor function in patients with a mild to moderate paresis, allowing additional voluntary muscle recruitment during FES‐assisted grasping. Therefore, all aforementioned objectives were also analyzed across different impairment levels. Subgroup analyses between different severity levels or the presence and absence of spasticity were performed by means of independent *t*‐tests or Mann–Whitney *U* tests. A mixed factor ANOVA was applied in a 2 (mildly/moderately vs. severely impaired) × 2 (initial vs. end grip force) design to test whether impairment severity showed an interaction with the force drop.

Data was processed and visualized using Matlab (MathWorks, USA) and inferential statistical analyses were performed using SPSS Statistics (IBM, USA). The alpha level was set to 0.05.

## 3 Results

### 3.1 Participants

**FIGURE 2:**
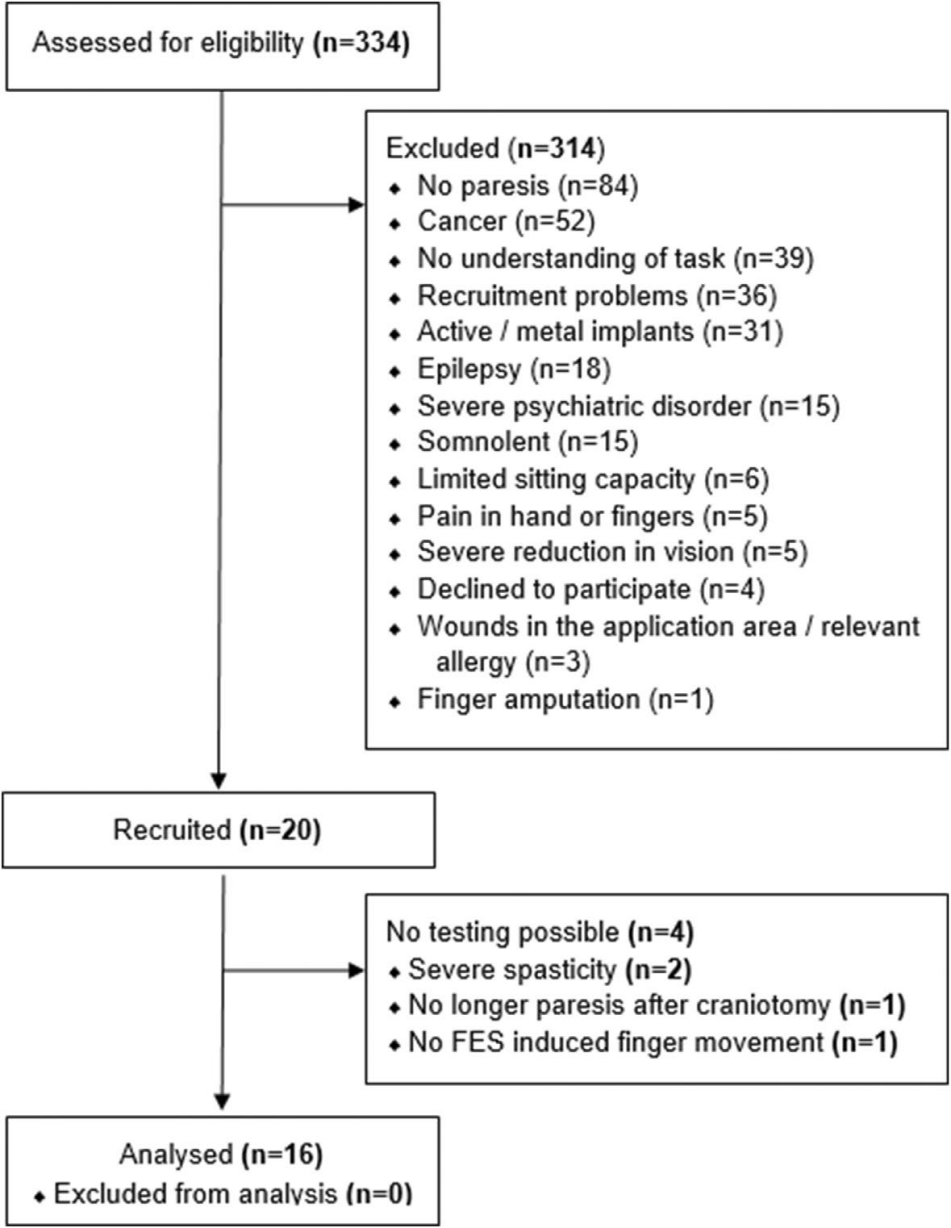
Flowchart of the recruitment process outlining the number of patients throughout the steps of screening, recruiting, and analyzing participants.

The following flowchart (Figure 2) reports the number of patients at each stage of recruitment.

Demographic and clinical data of all 16 patients who completed the experiment and were eligible for analysis is reported in Table 1.

**TABLE 1:** Demographic and clinical data of the study population.

Parameter	*M* (SD), min–max; or *M*, *n*
Age (years)	67.2 (14.7), 35–89
Sex (male/female)	9/7
Type of stroke (ischemia/hemorrhage/both)	11/4/1
Side of paresis (left/right)	10/6
Time after stroke (months)	3.4 (3.3), 0.8–13.9
Modified Ashworth Scale—Finger flexors (0/1/1 + /2/3)	0.6, 10/2/2/2/0
Modified Ashworth Scale—Finger extensors (0/1/1 + /2/3)	0.4, 12/2/1/0/1
Medical Research Council Scale—Wrist flexor (0/1/2/3/4)	1.9, 2/4/4/5/1
Medical Research Council Scale—Wrist extensor (0/1/2/3/4)	1.4, 4/6/1/5/0
Maximum grip strength (kg)	
Paretic hand	6.3 (6.5), 0.0–24.1
Non‐paretic hand	26.4 (10.0), 11.5–49.7

The following results on grip force metrics and the fatigue analyses are summarized in Table 2 at the end of the Section 3.

**TABLE 2:** Summary of results.

Grip force parameter			Subgroup analysis			
1. Grip force output						
12.7 ± 14 N			Mild‐moderately impaired (*n* = 5)			*p* = 0.023
			22.3 ± 18.6 N			
			Severely impaired (*n* = 11)			
			6.9 ± 6.0 N			
2. Grip force duration						
41.4% of repetitions ≥ 2 s			Mild‐moderately impaired (*n* = 5)			—
			37.4% of repetitions ≥ 2 s			
			Severely impaired (*n* = 11)			
			43.9% of repetitions ≥ 2 s			
Median transportation time: 1.8 s (IQR: 1.2–2.3 s)						
3. Object release						
Median release ratio: 1.6% (IQR: −0.6% to 12.3%)			Non‐spastic (*n* = 10)			*p* = 0.001
			0.5% (IQR: −0.7% to 8.6%)			
			Spastic (*n* = 6)			
			3.4% (IQR: −0.1% to 16.7%)			
Correlation with # of repetitions within block *r*(820) = −0.058, *p* = 0.068			Non‐spastic (*n* = 10)			—
			*r*(503) = −0.087, *p* = 0.050			
			Spastic (*n* = 6)			
			*r*(317) = −0.081, *p* = 0.149			
Correlation with # of repetitions across blocks: *r*(820) = −0.075, *p* = 0.035						
4. Fatigue						
Average force drop across blocks: −8.6% ± 23.5%			Mild‐moderately impaired (*n* = 5)			—
			−13.8% ± 21.5%			
			Severely impaired (*n* = 11)			
			−5.5% ± 25.1%			
Average force drop within block:			Mild‐moderately impaired (*n* = 5)			*p* ≤ 0.001 (severity × block)
			−11%	−18%	−5%	
Block 1 −3.1%	Block 2 −4.7%	Block 3 +2.9%	Severely impaired (*n* = 11)			
			+4%	+2%	+3%	

### 3.2 Requirements for Grasping Assistance—From Grip Force Production to Stability and Release

The first requirement for the assistive use of FES during object manipulation to be tested is the production of sufficiently high grip forces to lift an object. This requirement includes stimulator characteristics (e.g., ability to trigger forceful muscle contraction, comfort) as well as patient characteristics (e.g., voluntary strength).

**FIGURE 3:**
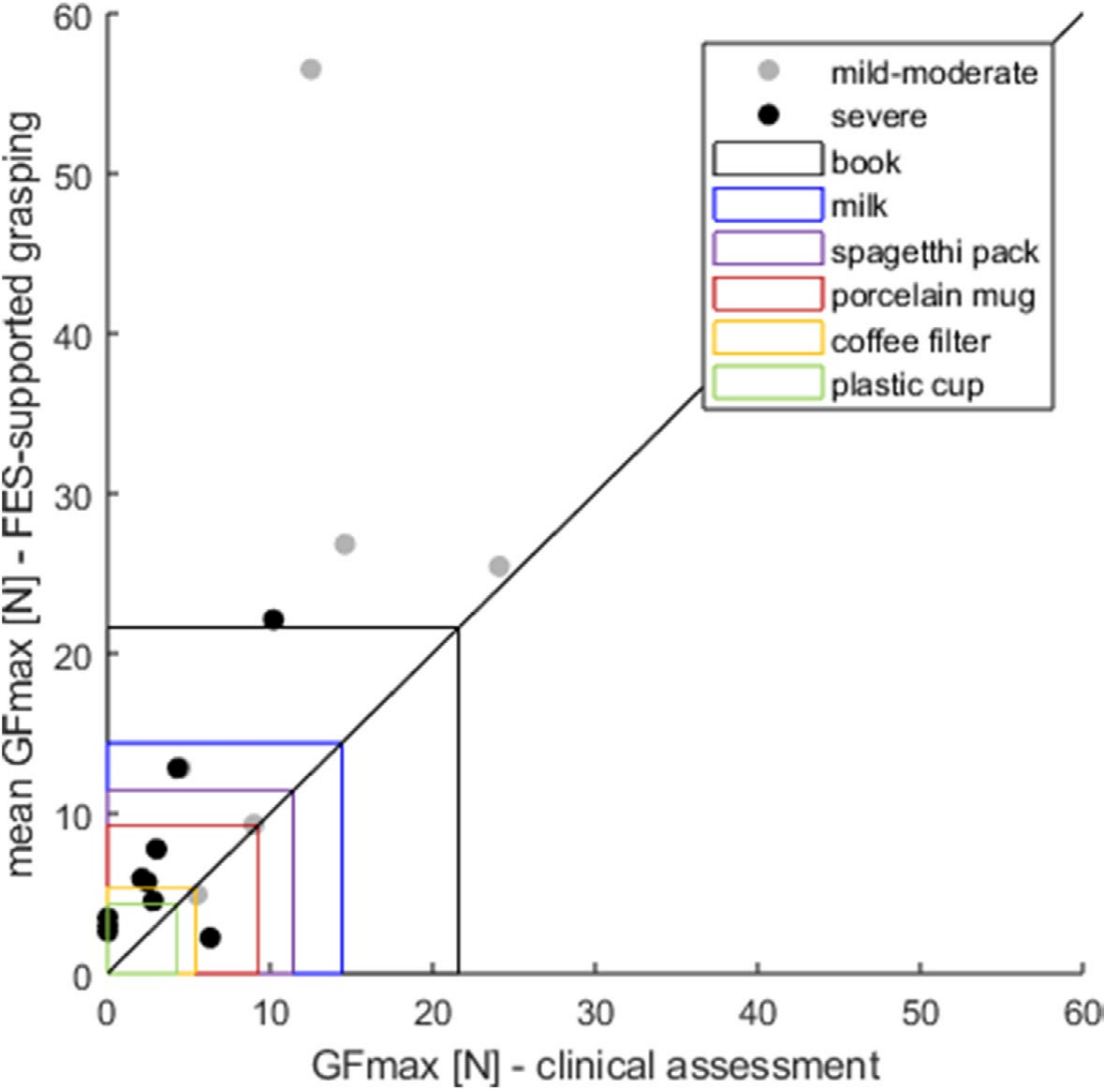
Patients' maximum grip force averaged across FES‐supported repetitions against their maximum unsupported grip strength in the clinical assessment. Severely impaired patients are represented by black dots, mildly/moderately impaired by gray dots. As a reference, colored rectangles indicate the minimum required peak grip force to lift ADL objects of the following weights: Book—805 g, milk—1060 g, spaghetti pack—526 g, porcelain mug—270 g, coffee filter—154 g, plastic cup—70 g [37]. [Color figure can be viewed at wileyonlinelibrary.com]

GFmax was averaged for each patient across repetitions in the three blocks and is visualized in Figure 3, compared against the patients' unsupported grip strength. During FES‐assisted grasping, patients applied an average GFmax of 12.7 ± 14.0 N (median 6.9, IQR 3.7–19.3 N), ranging between 2.1 and 55.7 N. Thus, all patients produced enough force to lift at least one reference object, and the FES‐assisted grasps of four patients were on average strong enough to lift the full list of objects. Mildly/moderately impaired patients produced, with a mean of 22.3 ± 18.6 N (median 18.6, IQR 8.2–34.0), a significantly higher GFmax than severely impaired patients with a mean of 6.9 ± 6.0 N (median 5.0, IQR 2.9–9.0, *Z* = −2.3, *p* = 0.023).

One mildly/moderately impaired subject showed a very positive response to FES assistance and therefore much higher grip force than in the clinical unsupported strength assessment. This patient was known to therapists at the clinic for the patient's unwillingness to make an effort, so the patient's lack of effort in the unsupported grip strength assessment could potentially bias their result. Thus, the high increase in grip force during the FES‐assisted task could be explained by the patient not putting full effort into the clinical assessment but engaging more in the gaming task with visual feedback on performance. There are two patients that produce, with 4.9 ± 0.3 N and 2.2 ± 0.6 N, slightly less grip force when assisted by FES than in the clinical assessment. For one of these patients, it was very hard to stimulate the flexors due to a feeling of discomfort at higher intensities.

As a second requirement, the ability of the patient, assisted by FES, to sustain a sufficient grip force for transportation was evaluated. In 86% of all repetitions performed by the whole study population, the lower boundary of the target grip force window was reached. Within these repetitions, the median transportation time was 1.8 s (IQR 1.2–2.3 s). In 41.4% of these repetitions, the force was maintained long enough for transportation (≥ 2 s [25]). Severely impaired patients maintained the target force for at least 2 s in 43.9% of repetitions, while mildly/moderately impaired patients had a transport time of at least 2 s in 37.4% of repetitions.

The third requirement to be tested was whether hypertonus was hindering object release during the stimulated hand opening part of a grasping sequence. The median release ratio was 1.6% (IQR: −0.6%–12.3%). However, in patients with spasticity, the median release ratio was significantly higher than in patients without spasticity (3.4% (IQR: −0.1%–16.7%) versus 0.5% (IQR: −0.7%–8.6%), *Z* = −3.45, *p* = 0.001). There was a weak negative correlation with statistical significance between release ratio and repetition number (1–60 (total number of analyzed repetitions: 820)). This suggests that the more repetitions performed, the smaller the grip force ratio that was applied during stimulation of extensors (*r*(820) = −0.074, *p* = 0.035). Similarly, the number of repetitions within one block (1–20, values averaged across blocks) tended to be significantly associated with a smaller release ratio (*r*(820) = −0.058, *p* = 0.068), particularly in patients without spasticity (*r*(503) = −0.087, *p* = 0.050), but not in patients with spasticity (*r*(317) = −0.081, *p* = 0.149). This might indicate that patients without spasticity improve their timing of object release over time, but patients with flexor spasticity cannot.

### 3.3 Requirements for Grasping Assistance–Repetitive Grasping at Required Grip Force

**FIGURE 4:**
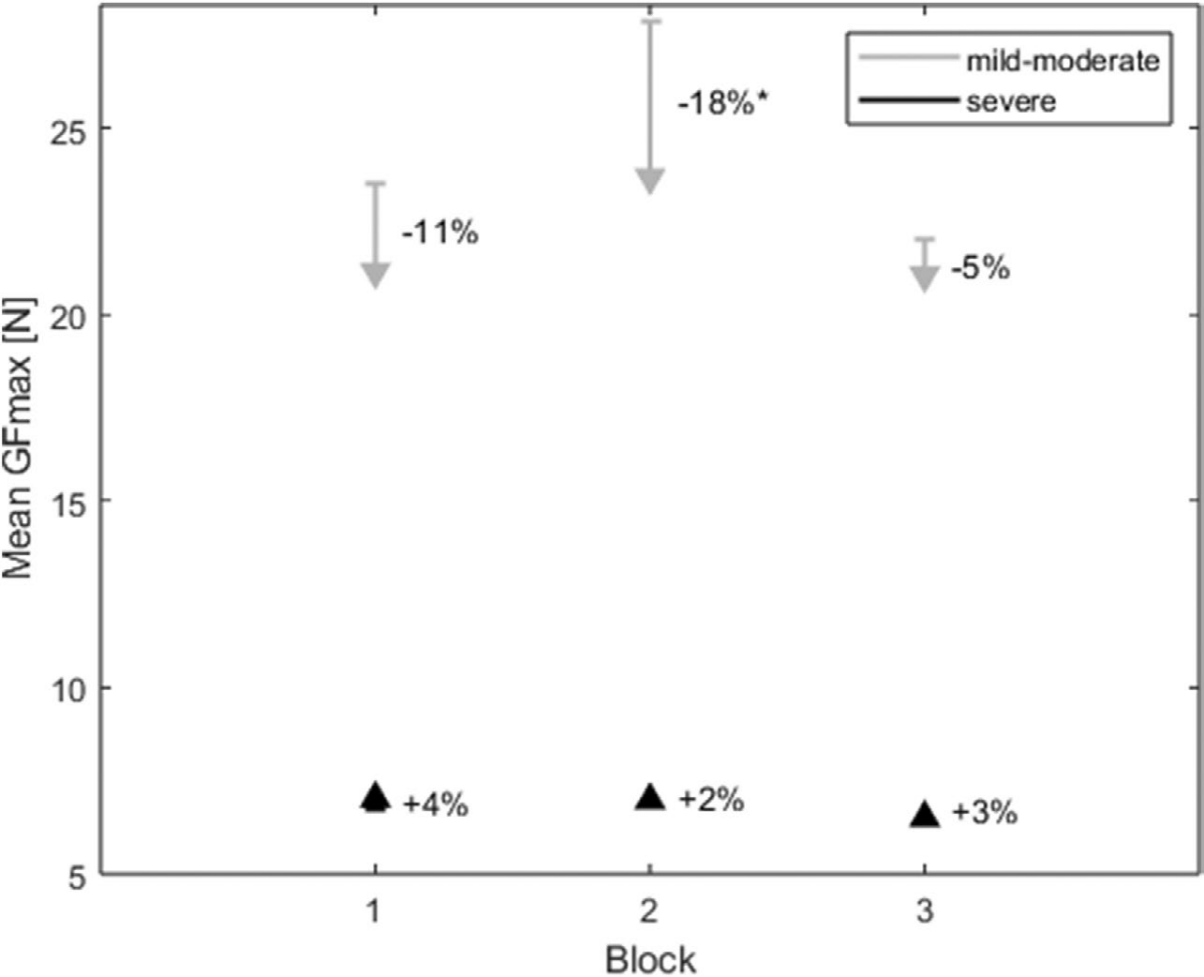
Force drop from initial GFmax to end GFmax for each block, separated by severity (*n* = 5 mild–moderately impaired patients, *n* = 11 severely impaired patients). *indicating a statistically significant force drop (*p* < 0.05).

Considering FES‐induced muscle fatigue, it was tested whether GFmax can be reached across repetitions. There was an average force drop across all 60 repetitions of 8.6% (SD 23.5%), showing a great range across patients from a decline by 52.6% to an increase by 43.4%. In patients with a mild–moderate hemiparesis, the maximum force dropped significantly by 13.8% on average (SD 21.5%, range −52.6% to 12.6%, *Z* = −2.15, *p* = 0.031). In patients with a severe hemiparesis, the force dropped by 5.5% (SD 25.1%, range −36.6% to 43.4%) from the beginning to the end of the experiment, which was not statistically significant (*Z* = −0.61, *p* = 0.544, Figure 4). Even though there was a statistically significant force drop from the beginning to the end of the experiment especially in mildly/moderately impaired patients, this decrease in GFmax is not considered muscle fatigue since it did not exceed 30%, which has been considered the criterion for muscle fatigue [38, 43].

When analyzing the force drop across all 20 repetitions within one block, there was a statistically significant decline in GFmax (*F*(1, 45) = 10.27, *p* = 0.002), with 3.1% in the first block, 4.7% in the second block, and an increase of 2.9% in the last block. There was a significant interaction between the change in GFmax and the severity of the paresis (*F*(1, 45) = 14.08, *p* < 0.001, Figure 4).

## 4 Discussion

### 4.1 Summary of Results and Relation to Previous Studies

This study evaluated requirements for the use of FES in assisting object manipulation including (i) forceful grasp production to lift objects, (ii) sustaining this force to transport objects, and (iii) successful extensor stimulation to release the object. Furthermore, the potential drop in grip force over time was evaluated because it could have implications for the effects of muscle fatigue on repetitive grasping in daily life.

Using FES, the median produced grip force was high enough to lift objects which are required for eating and drinking (e.g., plastic cup, porcelain mug). One quarter of patients produced grip forces exceeding 20 N, which is considered sufficient for manipulation of daily‐life objects [37]. However, it is important to note that the comparison between the generated force level and the force produced by healthy individuals lifting daily‐life objects is theoretical. Indeed, in the present study, patients did not actually lift these ADL objects. In this analysis, the circumference of objects was not considered, although it is known to have an effect on the required grip force (e.g., a wider grip span leading to a decrease in grip force [44]). Similarly, the impact of varying object surface texture was not tested, even though texture‐dependent friction between fingers and the surface of objects critically determines the necessary grip forces [45, 46]. Thus, generalizability to ADL objects is limited by the fact that patients might not have been able to produce the same grip force when grasping objects with a wider grip span or a slippery surface. Additionally, the reference values were taken from a younger study population compared to the tested patients. With higher age, higher grip forces are applied when lifting an object, potentially due to increased skin slipperiness in the elderly [47]. Thus, future trials should aim to evaluate whether FES‐assisted grip forces are actually high enough to lift these reference objects in a daily‐life scenario. Nevertheless, comparing the produced grip force values with the ones required to lift typical ADL objects provides a clearer interpretation of the potential of FES‐assisted grasping.

With an average of 7 N, patients with a severe hemiparesis applied less grip force than patients with a mild–moderate hemiparesis. Since the severely impaired patients were less able to voluntarily add grip force (mean MRC of finger flexors: 1.3), this value can be considered as FES‐produced force. This patient group is characterized by paretic muscles, which have not been volitionally activated for a longer period of time. Being able to produce a grip force of 7 N in patients with a severe hemiparesis is thus rated as a positive outcome of the stimulation.

There was a force drop from the first to the last three repetitions in mildly/moderately impaired patients but not in severely impaired patients. This finding suggests that the observed force drop in the group of patients with residual grip force was rather a consequence of a decrease in volitional force than of FES‐induced muscle fatigue. A decrease in force might be caused by a decline in motivation and by cognitive factors, since cognitive deficits were found to be related to motor performance in tracking tasks [42]. Most surprisingly, this force drop did not exceed the threshold determining muscle fatigue. Furthermore, although we expected to detect FES‐induced muscle fatigue due to the aforementioned physiological principles underlying FES, we did not find a force drop in severely impaired patients across repetitions. Indeed, previous work has reported muscle fatigue as a consequence of FES, but in these studies, continuous isometric grasping was investigated [43, 48]. This was not possible in the present study as the stimulation time had to be limited to 3 s. However, repetitive grasps have higher relevance for household ADLs than the isometric production of maximum force, since most of the ADLs around the household are performed within 2 min [49] and consist of subtasks involving different types of grasp sequences [50].

The median transport time was very close to the reference transport time of 2 s, which is typically required for individuals after stroke to carry objects from one point to another [25]. This shows that patients were able to sustain force while receiving FES assistance. When analyzing the data considering a strict 2 s transport time, the target grip force was maintained in less than half of the repetitions (40%). However, this does not mean that the patients could not transport any object in the remaining 60% of repetitions since the transport time refers to the time above the set target force. Patients might be able to better control lower grip forces (or transport lighter objects), especially since patients after stroke show better force control at lower grip force values, that is, best force control at 30% of their maximal voluntary contraction [51]. Similarly, as for GFmax, grip force stability was more consistent across repetitions in severely impaired patients (maintaining the target force for at least 2 s in 43.9% of repetitions) compared to mildly/moderately impaired patients (in 37.4% of repetitions). Again, this is an indicator that the results of severely impaired patients reflect the patients' capacity to produce force with the assistance of the stimulator, while there are additional influencing factors in mildly/moderately impaired patients, who were able to add voluntary force during the experiment, making the data more variable.

Regarding the patients' ability to release an object while receiving extensor stimulation, it was observed that patients with spasticity applied grip force during the hand opening period, while patients without spasticity did not. Patients with flexor spasticity applied force, even if it was only a small amount of force, during the extensor stimulation compared to their grip force during flexor stimulation (median release ratio: 2.5%, maximum release ratio: 16.7%). Thus, some patients with spasticity were not able to release the sensorized object, even when receiving FES assistance for hand opening. Furthermore, patients with spasticity did not release the object with a better timing during stimulation of extensors over time, but patients without spasticity did.

### 4.2 Limitations

The clinical trial was conducted in a laboratory, which created an artificial environment. This might have caused pressure and uncertainties for the participants, as the laboratory was an unfamiliar environment and they were conscious of being part of an experiment. This led to the exclusion of one participant with spasticity, who could generally perform FES‐assisted grasping therapy as part of the clinical treatment but was unable to release the flexor muscle tone upon entering the laboratory room. The patient was not able to grasp the sensorized object and perform the experiment. According to the subject's feedback, they felt increased tension and pressure when in the laboratory.

Furthermore, the stimulation intensity was not changed during the experiment with the aim to keep the grasping protocol consistent and steady and thereby reduce confounders on the fatigue analysis. However, patients with flexor spasticity might have profited from a decrease in flexor stimulation intensity and an increase in extensor stimulation intensity in order to counteract the exacerbation of muscle tone.

When investigating the development of muscle fatigue during repetitive FES‐assisted grasping, grip force was chosen as an evaluation parameter. Fatigue‐induced physiological changes (e.g., frequency change in electromyography [EMG]) precede alterations in the force level [52] and are less biased by the patients' effort. Especially in mildly/moderately impaired patients, it would have been interesting to additionally analyze EMG data. A combination of EMG and grip force data could help to distinguish whether the source of the grip force decline was due to physiological changes in the EMG data or rather due to other factors like attention or motivation. Nevertheless, fatigue is defined as failure to maintain force [52] and, from a therapeutic point of view as well as in daily‐life situations, a decline in grip force is more relevant than physiological changes.

As a general limitation, the nature of this study was largely theoretical as a sensorized object was grasped instead of real‐world objects. On the one hand, this has the advantage of providing FES‐assisted grip force metrics and information on muscle fatigue. On the other hand, the study's immediate applicability to practical real‐world ADL scenarios is restricted. Referring to normative data of non‐paretic grasping throughout the result section, however, should increase the interpretability of results (i.e., normative force values of different ADL objects, transport time of an object, threshold to define muscle fatigue). Nevertheless, the actual grip force behavior in a real‐world ADL scenario remains to be tested.

### 4.3 Future Research

Despite these limitations, this study is aligned with other scientific efforts to allow long‐term home‐based use of FES technology to assist upper extremity function. At this early stage of the exploration of orthotic FES applications, the focus was to establish performance metrics and allow the delivery of stimulation that is optimized to produce maximal force while reducing fatigue.

In subsequent work, the study environment should be shifted from the laboratory setting to a real‐life scenario in order to validate the conclusions of this trial for FES use in real‐life applications. Since grip force values in this trial were related to reference values of object manipulation from the literature, future studies should include real objects to further explore the assistive potential of FES during ADLs. Different types of grasps that are frequently applied during ADLs were identified [50], requiring variable grip types. Depending on the grip span, the maximum grip force that can be applied will vary, with a wider grip span leading to a decrease in grip force [44]. This emphasizes the need for further investigations with different sized real‐world objects. Grip force could be measured by flexible force‐sensors placed over the fingers [37] or a sensorized glove [44]. Lastly, during typical grasping in healthy individuals, grip force adaptation is used to scale the grip force efficiently. Whether an economic grip force scaling is possible while receiving FES assistance should be investigated in future trials.

## 5 Conclusion

All patients were able to produce grip force during FES assistance and maintained the grip force level across repetitions, without physiologically meaningful fatigue. The grip forces produced by the patients were similar to those found in real‐life object handling in healthy subjects and very close to the reference values required by stroke patients for object transportation. In addition, the repetitive grasping did not lead to a significant muscle fatigue. This grip force produced while having no relevant fatigue highlights the assistive potential of FES in ADL. However, these effects need to be verified for grasping of real‐life objects.

## Author Contributions

Chiara Höhler: conceptualization, data curation, formal analysis, investigation, methodology, visualization, writing – original draft. Satoshi Endo: formal analysis, software, writing – review and editing. Joachim Hermsdörfer: conceptualization, methodology, supervision, writing – review and editing. Lucille Cazenave: formal analysis, visualization, writing – review and editing. Hossein Kavianirad: conceptualization, software, writing – review and editing. Klaus Jahn: investigation, data curation, resources, writing – review and editing. Carmen Krewer: conceptualization, funding acquisition, methodology, project administration, supervision, writing – review and editing.

## Ethics Statement

This study was performed in line with the principles of the Declaration of Helsinki. Approval was granted by the Ethics Committee of Ludwig‐Maximilians University Munich (25.05.2021/No 21‐270).

## Consent

All procedures followed were in accordance with the ethical standards of the responsible committee on human experimentation (institutional and national) and with the Helsinki Declaration of 1975, as revised in 2000 (5). Informed consent was obtained from all individual participants included in the study or their legal representatives.

## Conflicts of Interest

The authors declare no conflicts of interest.

## Data Availability

The datasets generated during and/or analyzed during the current study are available from the corresponding author on reasonable request.
